# QEEG findings in nonsyndromic sagittal craniosynostosis

**DOI:** 10.1038/s41598-024-51858-2

**Published:** 2024-01-14

**Authors:** Tymon Skadorwa, Jolanta Strzelecka

**Affiliations:** 1 Department of Pediatric Neurosurgery, Bogdanowicz Memorial Hospital for Children, 4/24 Nieklanska St., 03924 Warsaw, Poland; 2 Department of Descriptive and Clinical Anatomy, Medical University of Warsaw, 5 Chalubinskiego St., 02004 Warsaw, Poland; 3 Department of Pediatric Neurology, Medical University of Warsaw, 63A Żwirki i Wigury St., 02091 Warsaw, Poland

**Keywords:** Brain, Paediatric neurological disorders

## Abstract

Despite the undertaken treatment, children with nonsyndromic sagittal craniosynostosis (NSC) are burdened with problems with speech development, visuospatial and other cognitive deficits. The electroencephalographic assessment has not influenced the diagnostics and treatment strategy of craniosynostosis so far but the introduction of quantitative EEG (QEEG) protocols renewed an interest in the functional aspect of this disease. In this study we retrospectively assessed the QEEG records of 25 children with NSC aged 1–18 months (mean age 9.62 months) before and after surgery. In each case, the amplitude, interhemispheric (ICoh) and intrahemispheric (HCoh) coherence indices were calculated. Obtained data were compared to age-matched control group of 25 normocephalic children. Children with NSC presented significantly lower values of amplitudes and intrahemispheric coherence in occipital, posterior parietal and posterior temporal regions than normocephalic children. The values of amplitudes, ICoh and HCoh in pre- and postoperative QEEG records mostly remained unchanged, with a slight improvement in HCoh in centro-parietal area. These findings suggest that NSC children present their own QEEG profile. The operative treatment improves an intrahemispheric connectivity, but there still exists a significant difference in the occipitotemporal, frontotemporal and centro-frontal areas, which may be considered as a functional substrate of reported speech and neurocognitive problems. QEEG findings in nonsyndromic sagittal craniosynostosis.

## Introduction

Developmental deformations of the skull in infants, especially those resulting from premature fusion of cranial sutures, are an area of a multidisciplinary interest^1,2^. Skull deformities and associated developmental problems constitute a hallmark of single suture synostoses, from which nonsyndromic sagittal craniosynostosis (NSC) is the most common type, estimated for 2–3:10,000 live births^1,3^.

Premature sagittal synostosis leads to an elongation of the skull in sagittal axis with a simultaneous constriction in transverse axis, often with compensatory changes, such as frontal and/or occipital bossing, or a retroorbital depression^4^. This cranial deformation affects the child's social and emotional development, making sagittal synostosis not only a surgical problem, often requiring an additional neurologic investigation and psychosocial support^1,5,6^. Children with NSC have normal IQ but in 7–37% they present verbal or language problems, visuospatial deficits and other cognitive delays^7,8^. The incidence of epilepsy among children with craniosynostosis is estimated at 5%, with such independent risk factors as brain compression, obstructive sleep apnea and hydrocephalus^9^.

An electroencephalographic interest in craniosynostosis dates back to 60’s of the XXth century. Few studies from that period focused mainly on the search for epileptiform discharges in EEG^10–12^ and hypothesized a predisposition to epilepsy in children with a craniosynostosis. The introduction of quantitative EEG (QEEG) protocols enabled the computational analysis of EEG records^13^. It is based on objective parameters, such as the amplitude, recorded in each channel, and coherence, a measure of phase synchrony between EEG signals^14,15^. The amplitudes and coherence indices have been studied in patients at various age^16–18^ and have been implemented in the diagnostics of neurologic and psychiatric diseases^19,20^ as well as in the assessment of functional connectivity within the cerebral hemisphere (intrahemispheric coherence, HCoh) or between the hemispheres (interhemispheric coherence, ICoh)^21,22^. HCoh may vary in conduction-affecting disorders (i.e., mild head injury has been found to raise its value), whilst dementia or Alzheimer’s disease is characterized by its decrease^23^. In turn, high ICoh values characterize the areas of well-developed connections^24^. Recent EEG studies on pediatric population revealed the usefulness of QEEG parameters in predicting language development, also in patients with craniosynostosis^25–27^.

### Clinical rationale for the study

The functional assessment of children with NSC has recently become one of the postulates of international collaborations aimed at patient stratification and clinical staging^28^. So far, the electroencephalographic assessment of children with craniosynostosis has been only a sporadic element of the management, not bringing significant changes to the diagnostic procedure and surgical technique. However, an increasing interest in the QEEG protocols in a developmental and clinical research has created the need of an objective, quantitative evaluation of the observed EEG phenomena, but to date any study was focused on children with craniosynostosis.

The primary goal of our study was to investigate the selected QEEG parameters (peak-to-peak amplitudes, HCoh and ICoh) in children with NSC compared to a group of healthy children in order to assess a relation between the cranial deformation and the electrical activity and connectivity in the brain. A rationale for using coherence and amplitude values was that a premature fusion of the sagittal suture, resulting in local skull constriction, may affect the power and hemispheric connectivity in children with NSC. These parameters can be easily obtained in every hospital setting, they are also reproducible and comparable with other papers. The secondary goal was to evaluate the effect of surgical treatment on the EEG records in patients with NSC.

## Materials and methods

### Study design

This retrospective study was performed on EEG records of patients with NSC treated in our institution between January 2018 and June 2020. The EEG was registered as an element of the diagnostic protocol of NSC in our institution and an informed consent was obtained from legal guardians of all patients. The study protocol was approved by the Bioethics Committee of Medical University of Warsaw (decision number AKBE/110/2021), and abides by the 1964 Helsinki Declaration and its later amendments or comparable ethical standards.

### Population

The group of children with NSC aged 1–18 months included 25 subjects (22 M, 3F). In all children a typical skull deformation (scaphocephaly) was diagnosed and a total or partial fusion of sagittal suture was evidenced by radiology. In none of the patients genetic aberrations, metabolic and immune system disorders, or a family history of craniosynostosis were reported. Mean age of the NSC group was 9.62 months, median age 6.83 months, standard deviation 4.83.

An age-matched control group was designed (mean age 9.42 months, median age 8.00 months, standard deviation 4.81). The control group included 25 infants (21 M, 4F) with normocephalic skulls and normal EEG records. In none of the controls seizures were observed and none of the patients received pharmacologic treatment.

### Method

In the study we retrospectively analyzed the QEEG data from preoperative examinations (group A, 25 cases) and compared them with postoperative records performed 3 months after the surgery (group B, 23 patients). Two patients were lost to follow-up. All patients were operated on with the same technique (strap craniectomies) by the same neurosurgical team. All surgeries were considered successful, with no complications. In 1-year follow up there was no need to repeat surgery in any patient. The obtained data were finally compared with a control group (group C).

Group A was also compared for age (below and above 6 months), pattern of sagittal suture fusion and differences in skull shape. The pattern of sagittal suture fusion included the indication of the fused 1/3 portion of the suture (A-anterior, M-middle, P-posterior, or a combination of above if more than one portion was fused). For the differences in skull shape a classification including 5 distinct types was used (dolichocephaly, leptocephaly, clinocephaly, bathrocephaly, sphenocephaly), as in the paper by Di Rocco et al.^29^.

### QEEG procedure

#### Recordings

The EEG was performed during physiologic sleep (NREM phase 2), in a quiet room with dim lighting. The examination was carried out with the use of Elmiko DigiTrack v. 14 device. QEEG data were obtained from 19 scalp electrodes placed according to 10–20 system. The impedance of each electrode was maintained below 20 kΩ. The sampling rate was 250 Hz, and the filtering range was 0.5–70 Hz.

A unipolar reference was used.

#### QEEG signal preprocessing

50 Hz notch filtering was performed to remove power frequency interference. The high-pass filter, with the − 3 dB cutoff the frequency of 0.5 Hz and the low-pass filter with the − 3 dB cutoff frequency of 70 Hz were selected. Physiological artifacts were removed using the software provided by the manufacturer.

#### QEEG parameters calculation

Power spectra for each lead were obtained with the Fast Fourier Transformation algorithm. The measured parameters included the amplitude, interhemispheric (ICoh) and intrahemispheric (HCoh) coherence indices calculated from an artifact-free epoch of 2 s duration.

The amplitudes were measured at 8 points for each side of the head: O1, P3, T5, C3, T3, F3, F7, Fp1 for the left hemisphere and O2, P4, T6, C4, T4, F4, F8, Fp2 for the right hemisphere. The amplitude was defined as the maximum peak-to-peak deviation of the signal within the epoch.

The coherence was defined by equation: Coh = (Sxy) 2/(Sxx × Syy), where Sxy, Sxx and Syy were cross-spectrum estimates of leads x and y, respectively. The HCoh values were computed for 24 intrahemispheric electrode pairs: O1–P3, O1–T5, P3–C3, P3–T5, P3–T3, C3–F3, C3–T3, C3–F7, F3–Fp1, T5–T3, T3–F7, F7–Fp1 for the left hemisphere and O2–P4, O2–T6, P4–C4, P4–T6, P4–T4, C4–F4, C4–T4, C4–F8, F4–Fp2, T6–T4, T4–F8, F8–Fp2 for right hemisphere.

The ICoh values were computed for 12 interhemispheric electrode pairs: O1–O2, P3–P4, C3–C4, F3–F4, T5–T6, T3–T4, F7–F8 and Fp1–Fp2.

### Statistical analysis

The calculated parameters were compared between the NSC group (A) and the control group (C) with the directional non-parametric Mann–Whitney rank-sum test using z-scores. The alternative hypothesis (control group parameters higher than in NSC group) was accepted if the results were considered significant (*p* < 0.05).

In an additional analysis the age groups were compared with Mann–Whitney rank-sum test. Multiple comparisons in relation to the pattern of sagittal suture fusion and the type of cranial deformity were performed using non-parametric ANOVA (Kruskal–Wallis test) with Bonferroni correction in order to avoid false significant results (an adjusted *p* value for the pattern and shape groups was set at *p* = 0.008).

The effect of surgery on QEEG was investigated by the comparison of parameters calculated for groups A and B (pre- and postoperatively). A non-parametric Wilcoxon signed-rank test was used for the analysis with *p* value < 0.05 considered significant.

The statistical analysis was performed with TIBCO Data Science/Statistica software by StatSoft Europe, version 13.3 PL for Microsoft Windows 10 Pro.

## Results

### NSC group versus control group in terms of amplitudes and coherence

**Figure 1:**
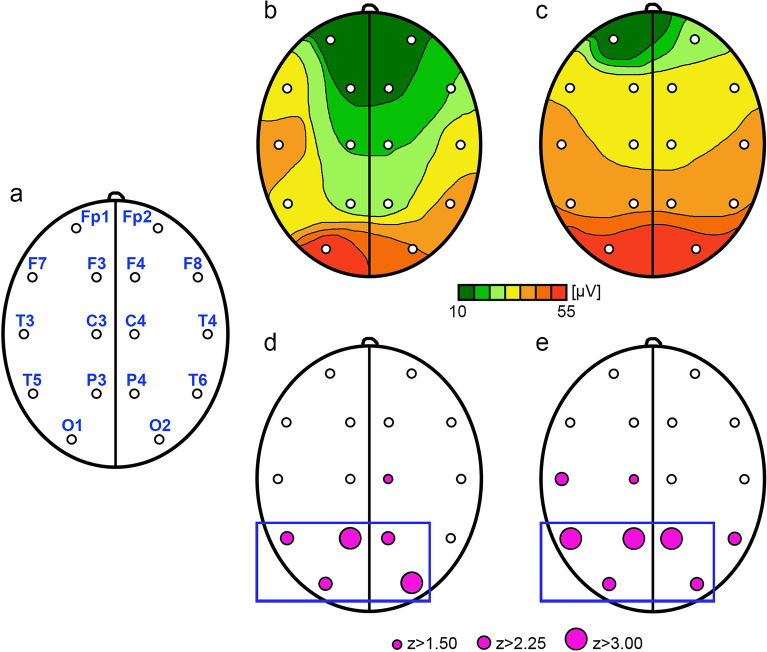
The location of electrodes (**a**), amplitude heatmaps (**b**, **c**) and amplitude differences between groups A–C (**d**) and B–C (**e**). Color maps show distinct distribution of amplitudes in NSC patients (**b**) and control group (**c**). Purple dots indicate EEG leads where the differences between groups were statistically significant; dots size reflect z-scores. Blue frame highlights the leads where the differences remained significant despite the surgery.

**Figure 2:**
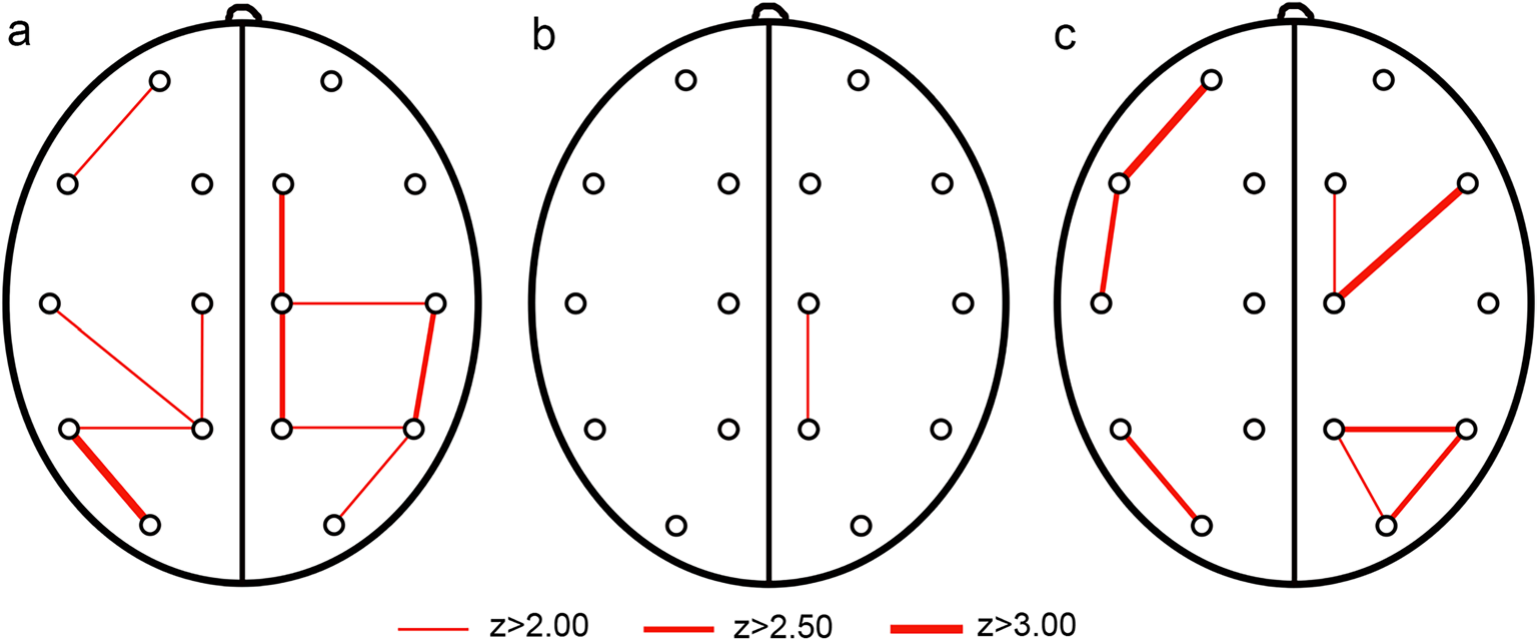
The differences in HCoh between groups A–C (**a**), A–B (**b**) and B–C (**c**) and their sizes reflect z-scores.

The amplitude was calculated for all leads. The amplitude values are presented in Table 1. Significantly lower amplitudes were found in leads O1, O2, P3, P4, T5 and C4 in the group of children with NSC (Table 1, Fig. 1). In terms of interhemispheric coherence, no differences were observed between groups A and C (Table 2). However, significant differences in intrahemispheric coherence were found, mainly in the occipital, posterior parietal and posterior temporal areas (Table 2, Fig. 2).

**Table 1:** The values of amplitudes according to leads placed over the left and right cerebral hemisphere.

[µV]	Group A (NSC preop)		Group B (NSC postop)		Group C (control)	
	Mean	SD	Mean	SD	Mean	SD
Left hemisphere						
O1	44.57	61.59	31.72	22.81	50.09	23.32
P3	21.78	13.16	22.98	12.18	35.63	13.95
T5	28.13	16.41	27.43	20.56	41.38	20.78
T3	31.45	21.37	25.85	17.59	35.48	14.22
C3	24.42	14.35	21.48	12.43	28.23	14.32
F3	17.21	9.00	20.48	12.11	24.51	23.59
F7	27.81	14.89	22.55	10.91	27.56	25.02
Fp1	16.18	7.80	14.81	9.98	16.05	11.05
Right hemisphere						
O2	31.96	31.48	32.12	23.46	51.72	28.32
P4	22.58	13.41	22.57	18.48	33.93	15.10
T6	35.77	18.48	27.55	20.92	39.18	15.99
T4	29.44	15.92	28.06	21.54	32.38	16.68
C4	21.44	10.23	22.74	15.43	29.25	16.34
F4	19.68	11.22	20.81	12.06	27.34	20.20
F8	24.53	15.77	30.50	20.48	27.96	40.43
Fp2	17.24	9.65	21.51	11.73	23.34	33.16

*SD* standard deviation.

**Table 2:** The values of interhemispheric (ICoh) and intrahemispheric (HCoh) coherence in the studied population.

	Group A (NSC preop)	SD	Group B (NSC postop)	SD	Group C (control)	SD
	Mean		Mean		Mean	
Interhemispheric coherence (ICoh)						
O1–O2	0.6946	0.0717	0.6977	0.0966	0.7164	0.0834
P3–P4	0.5841	0.0776	0.5628	0.0765	0.6066	0.0701
T5–T6	0.5745	0.1048	0.8794	1.6226	0.5842	0.0548
T3–T4	0.5546	0.0698	0.5377	0.0552	0.5812	0.0696
C3–C4	0.5510	0.0897	0.5523	0.0720	0.5565	0.0914
F3–F4	0.5519	0.0821	0.5449	0.0954	0.5557	0.0823
F7–F8	0.5735	0.0804	0.5297	0.0672	0.6176	0.0981
Fp1–Fp2	0.5895	0.1167	0.5484	0.0836	0.6008	0.0926
Intrahemispheric coherence (HCoh)						
O1–P3	0.6387	0.0695	0.6398	0.0763	0.6577	0.0763
O1–T5	0.6245	0.0657	0.6295	0.0820	0.7041	0.0823
P3–C3	0.6208	0.0953	0.6399	0.1016	0.6896	0.1154
P3–T5	0.6651	0.0781	0.6899	0.0570	0.7248	0.0733
P3–T3	0.5915	0.0706	0.6143	0.0755	0.6491	0.0964
C3–F3	0.5937	0.1100	0.6190	0.1220	0.6251	0.1026
C3–T3	0.6285	0.1093	0.6487	0.0686	0.6464	0.0881
C3–F7	0.5856	0.1044	0.5671	0.0831	0.6024	0.0647
F3–Fp1	0.5693	0.1254	0.5572	0.1118	0.6099	0.1322
T5–T3	0.6555	0.0892	0.6718	0.0661	0.6848	0.0875
T3–F7	0.6427	0.0829	0.5985	0.0825	0.6791	0.0855
F7–Fp1	0.5817	0.1327	0.5519	0.0959	0.6446	0.0876
O2–P4	0.6375	0.0804	0.6244	0.0723	0.6700	0.0758
O2–T6	0.6601	0.0758	0.6288	0.0998	0.7114	0.0768
P4–C4	0.5985	0.0969	0.6917	0.1218	0.6826	0.0834
P4–T6	0.6597	0.0773	0.6495	0.0816	0.7121	0.0773
P4–T4	0.5805	0.1062	0.6086	0.0752	0.6193	0.0862
C4–F4	0.5802	0.1003	0.5783	0.0909	0.6434	0.0811
C4–T4	0.5981	0.0830	0.6057	0.0878	0.6492	0.1001
C4–F8	0.5733	0.0766	0.5441	0.0480	0.6026	0.0986
F4–Fp2	0.6230	0.0919	0.6138	0.1194	0.5877	0.1044
T6–T4	0.6096	0.0807	0.6572	0.0906	0.6598	0.0502
T4–F8	0.6162	0.0928	0.6095	0.0803	0.6275	0.0765
F8–Fp2	0.6317	0.0873	0.6269	0.0629	0.6341	0.1079

*SD* standard deviation.

### Amplitudes and coherence in NSC children in relation to age, pattern of sagittal suture fusion and skull shape

#### Age

In the studied population there were 12 children at the age below 6 months and 13 children above this age (4 children were older than 12 months). Mean amplitudes in most leads were higher in children older than 6 months (differences not statistically significant) but the only significant difference was observed in O1 lead (*p* = 0.0377, z = 2.0776) in favor of younger children. No differences were found in terms of interhemispheric coherence. In terms of HCoh, the differences were significant for P3–T3 (*p* = 0.0324, z = 2.1396) and C3–T3 (*p* = 0.0438, z = 2.0156). In both cases, higher values were noted in children at the age below 6 months. The amplitudes and coherence in children older than 12 months were not statistically different comparing to individuals aged 6–12 months.

#### Pattern of sagittal suture fusion

Four distinct pattern of sagittal suture fusion were identified: M (5 cases), MP (9 cases), AMP (10 cases) and AM (1 case). In the comparison of patterns, no significant differences in amplitudes were found. The highest ICoh values were observed for the MP pattern, but only in O1–O2 the values were significantly different from other patterns (*p* = 0.0060, H = 10.2261). There were no differences in intrahemispheric coherence.

#### Skull shape

Four types of cranial deformity were identified: sphenocephaly (10 cases), clinocephaly (8 cases), bathrocephaly (4 cases) and dolichocephaly (3 cases). The highest amplitude values were observed in dolichocephaly (not significant). No differences in terms of amplitudes and coherence indices were observed among the skull shapes.

**Figure 3:**
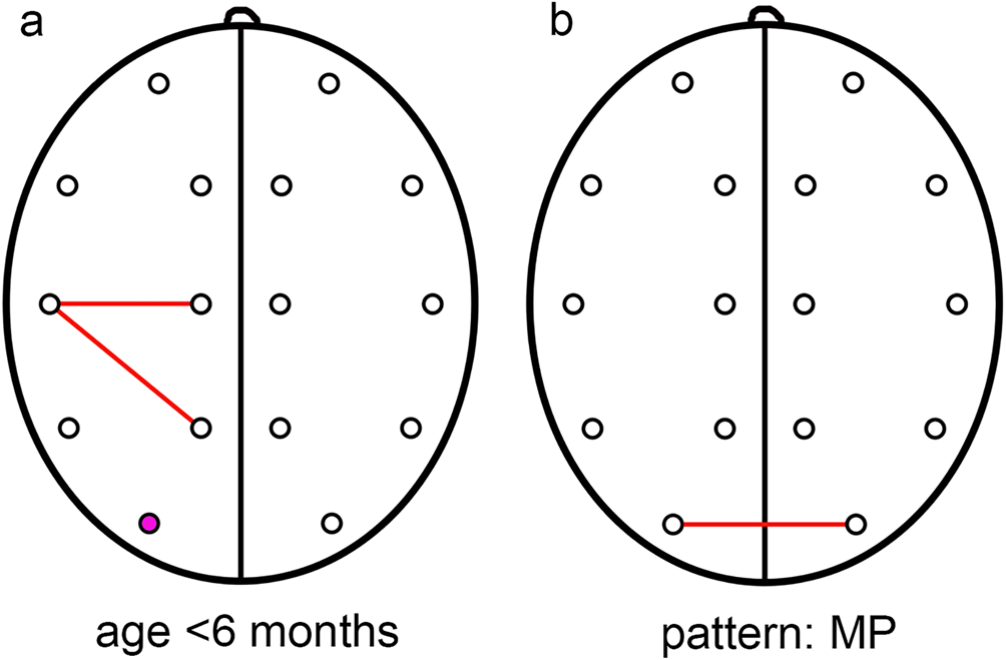
The differences in QEEG in NSC patients according to age (**a**) and pattern of sagittal suture fusion (**b**). A purple dot indicates an EEG lead where the difference in amplitude between groups was statistically significant. Red lines indicate significant differences in HCoh (**a**) and ICoh (**b**).

The differences depending on age and pattern of sagittal suture fusion are presented in Fig. 3.

### Amplitudes and coherence in NSC patients before and after surgery

In terms of amplitudes, only the T6 lead showed a significant difference between groups A and B (*p* = 0.0244, z = 2.2507). In the remaining leads, no significant differences were registered before and after the surgery (Table 1). Postoperative ICoh values were lower than preoperative (Table 2), but the only significant difference was in F7–F8 (*p* = 0.0244, z = 2.2507). In terms of intrahemispheric coherence, no significant differences were found between groups A and B (Table 2), with the exception of HCoh for P4–C4, which was higher after the surgery (*p* = 0.0157, z = 2.4156).

## Discussion

Electroencephalography in patients with craniosynostosis provides an interesting information about the brain functioning, even many years after the diagnosis^30^. Nevertheless, EEG is not typically included in the standard diagnosis of premature suture fusion, except in a few cases of syndromic craniosynostosis^31^. To date, no EEG pattern has been recognized as characteristic for craniosynostosis, despite the objective differences in brain structure resulting from this disease^32^. However, despite the implemented treatment, findings on cognitive functioning in NSC children still indicate considerable problems^1,33^, refocusing an interest of researchers on detailed EEG assessment with the use of objective parameters.

As shown by the results, children with NSC, despite normal EEG records and lack of epileptiform graphoelements, present some differences in the electroencephalographic profile comparing to normocephalic children. This is evident both in the amplitudes calculated bilaterally in occipital and posterior parietal areas, but also in HCoh, reflecting the condition of intrahemispheric connections.

The distribution of significantly lower amplitudes in children with NSC corresponds to typical areas of cranial constriction in posterior parietal and occipital regions and may be related to local brain compression, which was postulated by other authors^34^.

Amplitude values in NSC patients were not specific for the child’s age, although a trend to increase with age was observed. Despite various types cranial deformation and different patterns of sagittal suture fusion, the amplitude values did not differ significantly across our NSC population. Single differences observed in the age subgroups may suggest subtle alterations in occipital areas in NSC children comparing to healthy subjects.

Surgical procedure did not change the mean amplitudes in children with NSC. In our population, the only change observed was a decrease in the mean amplitude in right posterior temporal area. Nevertheless, significant differences in occipital, parietal and left posterior temporal areas were still observed postoperatively (Fig. 1). In addition, the noted differences in C3 and T3 leads do not seem to be related to surgery.

Concluding, despite showing some individual differences, we do not see significant variation in amplitudes between age groups, pattern of sagittal suture fusion or skull shape.

The situation is different with intrahemispheric coherence, which showed marked differences between the group of children with NSC and the control group. In the NSC group, the differences in connectivity clearly concerned the occipital, posteroparietal, posterotemporal and, to a lesser extent, centro-frontal areas, indicating the regions of significantly smaller intrahemispheric connectivity.

Interestingly, the implemented treatment did not significantly improve the intrahemispheric coherence in children with NSC and after surgery they still presented lower Hcoh values than the control group (Fig. 2). It seems that surgery allows some improvement of HCoh in the posterior temporoparietal and centro-temporal areas, but the decreased values still remain bilaterally in the area of the occipitotemporal junction, which might be considered as a microfunctional substrate of reported neurocognitive decline. An additional observation is the persistence of reduced HCoh values in frontotemporal and centro-frontal areas, which may be related to problems with speech development observed in children with NSC even after surgical treatment^35^.

Interhemispheric coherence did not differ between groups A and C, suggesting that NSC children do not differ from normocephalic children in terms of interhemispheric connectivity. It is worth emphasizing that surgery does not affect the ICoh and even contributes to a slight decrease in connections between the posterior frontal areas. It seems, however, that both this observation and the decrease of HCoh in frontal regions may reveal a problem that is possibly not sufficiently addressed in the surgical technique. Classic surgical methods (inverted "pi", strap craniectomies, barrel-stave osteotomies) and minimally invasive techniques (endoscopy-assisted suturectomies or spring-assisted surgery) do not primarily focus on the frontal and fronto-basal regions. Leaving these areas intact may promote further local constriction which to some extent may be associated with a decrease in the number of associations in frontal areas. In our opinion, however, these results, along with the existing clinical evidence, do not support the concept of an extension of the surgical field to these regions only due to the QEEG findings.

### Strengths and limitations of the study

The most important advantage of this work is a novel insight into the EEG recording in NSC children. In this paper we characterize not only the values of amplitudes in individual leads, but also the values of intrahemispheric and interhemispheric coherence. It was also possible to relate these findings to normocephalic children and to compare the impact of surgery on the calculated parameters.

However, this study has several limitations. The QEEG data were obtained from 19 scalp electrodes placed according to 10–20 system, which may be burdened with inaccuracies and spatial aliasing^13^. The system, however, is still widely used in research and in clinical practice ensuring comparability and repetitiveness^36^. In our opinion the findings should be validated in a setting based on higher resolution EEG techniques. The second limitation is the relatively small number of subjects, which means that the obtained data are rather illustrative and do not constitute clear guidelines in the diagnosis of patients with NSC. A larger number of children would make the observed trends credible. The third limitation is the fact that the presented QEEG data were not correlated with the neurodevelopmental data, mainly due to the age of our patients. Language assessment tests (such as Bayley scale version III) are less reliable in infants than in older children. In addition, the third version of Bayley scale was not yet validated in Polish population at the time of the study, therefore an electroencephalographic-developmental correlation is still one of our goals in the future.

## Conclusions

Children with NSC have their own unique EEG profile. The differences in relation to normocephalic individuals are visible mainly in the occipital, posterior parietal and posterior temporal regions. NSC patients achieve lower values of amplitudes and intrahemispheric coherence there, but interhemispheric connectivity seems not to be affected comparing to normocephalic subjects.

Surgical treatment does not change the EEG profile of NSC children—differences in amplitudes in the occipital, posterior parietal and posterior temporal regions are still visible after surgery. The operation improves intrahemispheric connectivity (associations), but there is a marked difference in the anterior temporofrontal and centro-frontal areas, which may be related to speech development difficulties and other neurocognitive disorders being a potential goal of future therapies targeted at coherence improvement (i.ex. neurofeedback).

Further investigation based on higher resolution EEG techniques is needed in order to validate these preliminary findings. A clinical correlation with speech and neurocognitive delay might promote the use of QEEG as a prescreening method of early diagnostics of future neurodevelopmental and, potentially, mental disorders.

## Data Availability

The data belong to Bogdanowicz Memorial Hospital for Children in Warsaw and are not available to share unless in the form included in the manuscript and supplementary materials. For additional data requests please contact Dr. Tymon Skadorwa at neurochirurgia@nieklanska.pl.
